# Systematic Repurposing Screening in Xenograft Models Identifies Approved Drugs with Novel Anti-Cancer Activity

**DOI:** 10.1371/journal.pone.0101708

**Published:** 2014-08-05

**Authors:** Jeffrey J. Roix, S. D. Harrison, Elizabeth A. Rainbolt, Kathryn R. Meshaw, Avery S. McMurry, Peter Cheung, Saurabh Saha

**Affiliations:** 1 BioMed Valley Discoveries Inc., Kansas City, Missouri, United States of America; 2 Charles River Discovery Research Services, Morrisville, North Carolina, United States of America; Clinica Universidad de Navarra, Spain

## Abstract

Approved drugs target approximately 400 different mechanisms of action, of which as few as 60 are currently used as anti-cancer therapies. Given that on average it takes 10–15 years for a new cancer therapeutic to be approved, and the recent success of drug repurposing for agents such as thalidomide, we hypothesized that effective, safe cancer treatments may be found by testing approved drugs in new therapeutic settings. Here, we report *in-vivo* testing of a broad compound collection in cancer xenograft models. Using 182 compounds that target 125 unique target mechanisms, we identified 3 drugs that displayed reproducible activity in combination with the chemotherapeutic temozolomide. Candidate drugs appear effective at dose equivalents that exceed current prescription levels, suggesting that additional pre-clinical efforts will be needed before these drugs can be tested for efficacy in clinical trials. In total, we suggest drug repurposing is a relatively resource-intensive method that can identify approved medicines with a narrow margin of anti-cancer activity.

## Introduction

Despite tremendous research efforts, one in four Americans will die as a result of cancer. The quest to identify novel treatments for cancer has spurred numerous, broad innovations in biomedical research, and cancer care is at the forefront of molecular medicine, where tailored diagnostics and drugs are used to precisely target the genetic basis of cancer [1]. Still, controversy exists as to whether the efforts and cost of cancer research and care have truly resulted in sufficient societal benefits; it is unclear when and how a victory in the war on cancer may be declared [2]–[4].

It has been suggested that novel therapeutics for many diseases including cancer may be found by exploiting medications that are already approved for use. [5] Several precedents for compound “repurposing” exist, and it is expected that the diverse pharmacology targeted by approved medications may have unknown, unexpected utility in diseases beyond the label indications for which those drugs are currently prescribed. As many approved drugs have a well-established history of safe dosing in broad populations, novel repurposing indications can likely be rapidly tested directly in human subjects, without the need for extensive preliminary safety assessments.

Given this potential value, we tested a broad collection of approved medicines dosed in combination with chemotherapy in mouse xenograft cancer models. While our unbiased screening and validation strategy identified approved drugs with combination chemotherapy potential, additional mechanistic and regulatory studies would likely be required before these agents could be assessed in clinical trials.

## Materials and Methods

### Animal Xenograft Studies

9 week old female athymic nude mice (Crl:NU(Ncr)-*Foxn1nu*, Charles River) maintained on standard light cycle and fed *ad libitum* water and NIH 31 diet were used for the studies. Tumor xenografts were initiated by implantation of 1 mm^3^ tumor fragments from source xenograft tissue maintained by serial transplantation. Tumor growth was monitored as the average size approached the target range of 80 to 120 mm^3^, and animals were then subsequently randomized to treatment cohorts. Tumors were measured in two dimensions using calipers, and volume was calculated using the standard formulas.

Temozolomide was administered orally, once-daily for the first five days of the study. Combination treatments were administered once daily using the indicated route and formulation; experimental drugs were dosed both during and after temozolomide administration to identify sensitization effects from both concurrent and follow-on exposure.

All animal husbandry, treatments and veterinary care were conducted by trained personal in AAALAC-accredited facilities; protocols and study conduct were subject to IACUC review and approval at Charles River.

### Statistical Methods, Screening Power Estimates

To capture the time-course data of xenograft studies, we employed log-rank Mantel-Cox survival analysis, where survival cutoff was defined by the emergence of a specific tumor burden on study. Following analyses of pilot studies, we determined that a 500 mm^3^ tumor volume cutoff adequately captured the therapeutic effects of increased temozolomide dose. Log-rank chi-square was used to calculate and report significance testing; effect sizes are reported as Mantel-Haenzel hazard ratios, where a protective ratio reflects the ability of the experimental treatment to delay or prevent tumor progression beyond 500 mm^3^. As discussed in results, choosing cut-offs greater than this threshold did not markedly influence results. 1000 mm^3^ were somewhat more variable, with systematic affect effects on statistical precision within and across groups; growth at 500 mm^3^ appeared more homogeneous.

For screening, typically 10 animals were assigned to receive 5 mg/kg temozolomide monotherapy, while most frequently 5 animals were assigned to combination treatment. Power calculations indicated this study design was adequate to discern a 2.6-fold reduction in the survival hazard-rate with 80% power at a significance level of 0.05 (derived using formulas contained in: http://www.cct.cuhk.edu.hk/stat/survival/Rubinstein1981.htm). As discussed below, the practical impact of uncovering effect sizes of this magnitude yielded observations where the experimental combination provided added efficacy equivalent to a 2-fold increase in the dose of temozolomide. In another light, our powering approach would typically yield the following: while all 10 animals treated with temozolomide monotherapy reached tumor burdens beyond 500 mm^3^ within 15 days, significant screen hits could be identified when no more than 1 of 5 animals in the combination cohort had a tumor burden more than 500 mm^3^ after 30 days of treatment and follow-up.

### Repurposing Library

A candidate list of approved drugs was compiled from DrugBank [6], the Therapeutic Targets Database [7], and the Merck Manual of Diagnosis and Therapy. Following assessments of cost and availability, gram-scale compound stocks were provisioned from multiple vendors. Dose formulations and routes appropriate for chronic dosing were identified by literature search. Drug targets and mechanisms were queried against the latest edition of the Therapeutic Targets Database. To-date, TTD categorizes 364 targets as “successful”, having been targeted by approved drugs. Our experimental set was mapped to 124 successful targets, or approximately 35% coverage. Our coverage estimate is conservative, as we ignored the one-to-many relationships for drugs that mapped to multiple targets; multiple targets of any drug were not double-counted if at least one of a drug's several targets was redundantly targeted by another drug.

### Temozolomide/Candesartan PK Interaction Study

Radio-isotope labeled temozolomide (4-methyl-^14^C-5-oxo- 2,3,4,6,8-pentazabicyclo [4.3.0] nona-2,7,9-triene- 9-carboxamide) was synthesized (Moravek) such that scintillation counting could assess the following: intact compound; active metabolites; as well as covalent adducts known to be formed following reactive metabolism of temozolomide in target tissues [8].

Dose interaction experiments were conducted in female nude mice bearing U87MG tumor fragment xenografts, as described above; animals had tumor burden of ∼400 mm^3^ at the time of dosing. Mice were administered either vehicle or candesartan i.p. for one day preceding combination dosing with C14-temozolomide. On the next day, a 5 mg/kg dose of C14-temozolomide was administered orally either alone or immediately following a dose of 10 mg/kg candesartan by i.p. administration.

At the time points indicated, tissues were collected and assessed for radio-isotope distribution by standard scintillography methods.

### Combination Tolerability Study

8–10 week old, healthy male C57Bl/6J mice were administered either temozolomide alone at 25 mg/kg, or together with combination agents at the indicated routes and dose levels. Following six days of combination dosing, temozolomide administration was discontinued, while the combination agents were administered for another 4 days. Animals were sacrificed at the indicated time points, and blood samples were analyzed for complete blood count parameters using standard hematological methods.

### Dose Range Response Study Background Information

Prescribing information labels were used to guide dose selection for dose-range response studies.

For risedronate (brand name: Actonel [9]), nonclinical 80-week carcionogenicity studies were performed in mice at 32 mg/kg/day. Using body-surface area normalization, the label estimates this is the dose equivalent of 6.5 times the maximum recommended daily dose in humans (30 mg/day), which is indicated for use in Paget's disease. Therefore, the screening and top dose-range finding level in our mouse experiments of 7.5 mg/kg/day is only slightly above the maximum recommended human daily dose (MHRD), based on body surface area normalization. The lowest mouse dose tested, 1.5 mg/kg/day, is largely equivalent to the lower human daily dose (5 mg/day) that is recommend in osteoporosis therapy.

For candesartan (brand name: Atacand [10]), nonclinical 104 carcinogenicity studies in mice were conducted at 100 mg/kg/day. The Atacand label estimates that this dose elicited systemic exposures in mice (on a AUC basis) that were approximately 7 times those achieved in humans following administration of the MHRD, 32 mg/day. Therefore, the screening and top dose-range finding level in our mouse experiments of 10 mg/kg/day were likely above the MHRD, on a AUC-normalized exposure basis, particularly given our initial dosing by i.p., rather than p.o. route. Mouse experiments at 2.5 mg/kg/day p.o. should be largely reflective of the low entry dose indicated for use in hypertensive adults (8 mg/day).

For terbinafine (brand name: Lamisil [11]), 28 month rat carcinogenicity studies were conducted at 69 mg/kg/day; this dose is stated to be 2 times the MHRD, based on AUC exposure comparisons. Our screening and dose-range studies in mice used i.p. administration, suggesting the 50 mg/kg/day dose by this route is likely well above the MHRD equivalent, given that the drug displays modest bioavailability across species [12], [13]. In support of this, 50 mg/kg oral doses in mice were previously shown to elicit peak systemic concentrations on the order of 1 ug/mL [14], which is equivalent to cMax observed in humans following a 250 mg dose. We did not pursue an oral dose-range finding study in our mouse models, given the poor responses observed following dose titration by i.p. route.

## Results

**Figure 1:**
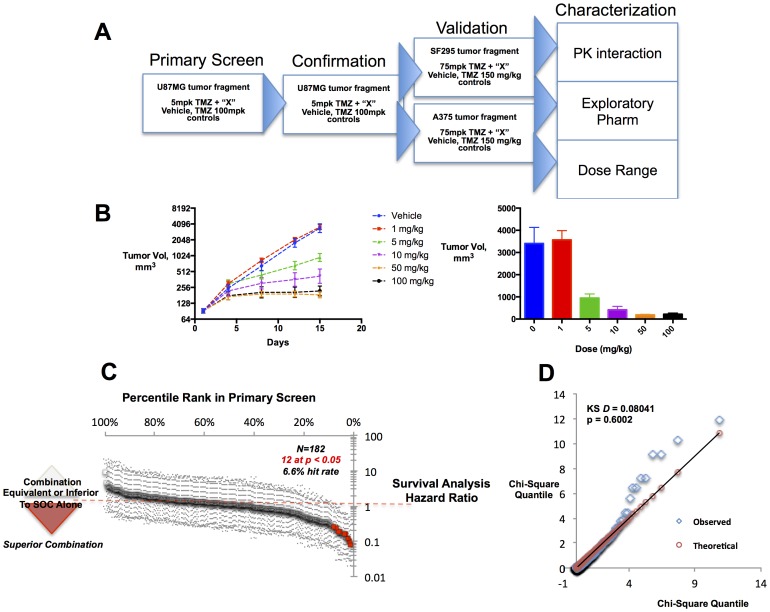
Experimental design, model development and screen results. A. Experimental testing funnel. Approved drugs were identified and re-confirmed in a screening model. Hits were further tested complementary models, and characterized in pilot pharmacology studies. B. U87-MG Screening Model Design and Characterization. In dose range-finding experiments, the dose level of 5 mg/kg of temozolomide was chosen as the partially effective dose that used in subsequent combination studies. C. Primary screen overview. The hazard ratios of approved drugs in survival analysis (500 mm^3^ tumor burden cutoff) are plotted by rank. Two-sided confidence estimates are shown, with significant hits (N = 12, Table 1) highlighted in red. D. Hit rate analysis. Experimentally observed survival analysis effect sizes (q) are plotted against the chi-square distribution; the Kolmogorov-Smirnov statistic confirms the null, that each derive from the same underlying distribution.

We designed a staged experimental testing strategy in order to screen, confirm and validate approved drugs that could have potential activity as combination chemotherapy agents (Figure 1A). First, we attempted a relatively rapid primary efficacy screen using low animal numbers (typically 10 control and 5 experimental animals per combination), testing each compound at a single dose level. Promising hits from the primary screen were re-tested in the same model to assess reproducibility. Reproducible hits were next tested in two additional xenograft models to test whether the findings were generalizable. Several compounds were tested in dose-response studies, as well as additional pilot studies aimed at characterizing the possible mechanistic activity behind each hit. Ultimately, our testing schema (Figure 1A) was designed to identify compounds that demonstrated reproducible, robust pharmacology worthy of rapid translation into clinical settings.

We established a model system and compound collection to survey a broad range of pharmacology in a clinically relevant setting. We focused on glioblastoma as a disease setting with high unmet clinical need. Our primary screen was conducted in the U87-MG glioblastoma-derived human cancer cell line; xenografts of this line were developed using tumor fragment serial passage methods. We confirmed this model was responsive to a standard-of-care chemotherapeutic for glioblastoma; the alkylating drug temozolomide elicited complete tumor responses in a majority of animals at a dose of 100 mg/kg. Based on prior pharmacology studies, we note the U87-MG model seems relatively treatment-refractory: exposures achieved near the complete response dose (AUC_0-inf_ ∼125 ug*hr/mL, data not shown) likely exceeded those obtained in humans when following standard dosing guidelines (AUC_0-inf_ ∼20 ug*hr/mL).

We assembled a diverse compound collection to test the majority of pharmacological mechanisms targeted by currently approved drugs. The approximately 1200 prescribed drugs target likely no more than 300 unique pharmacological mechanisms [15], [16]—we estimate our selected library of 182 compounds recapitulates approximately 35% percent of the pharmacology targeted by the human pharmacopeia (methods). On a practical basis, we relied on published literature to help chose dose levels and drug formulations. Crucially, dosing levels and administration routes were selected to obtain drug exposures near or beyond those achieved using doses that are maximally tolerated in humans (Table S1 in File S1). In short, we followed a “greedy” strategy that first probed supra-pharmacological effects in screening mode, followed by hit validation at exposures nearer the situation in everyday clinical use.

Several notable findings could be observed during our primary efficacy screen of the 182 compound set in the U87-MG model (Figure 1C, Table S1 in File S1). First, using experimental methods that were technically feasible and reasonably resource-effective, we could observe that temozolomide and approved drugs showed combination efficacy during relatively high-throughput screening. Twelve primary hits were identified that delayed the time needed to achieve palpable tumor burden in treated animals (Table 1). These hits exhibited efficacy in combination with temozolomide broadly comparable to what could be obtained by doubling the therapeutic dose of temozolomide (Figure 1C, Methods). Results were reproducible: in 10 of 11 experiments, a highly active dose of temozolomide (100 mg/kg) was highly significantly different from the moderate dose (5 mg/kg) used in combination tests, and showed modest statistical discrimination in the remaining study (data not shown). Encouragingly, the known chemotherapeutics docetaxel and etoposide showed efficacy in combination with temozolomide (Table 1). Also, the overall primary hit rate (6.6%) did not appear excessively high, which could suggest unsuitable effects due to experimental variability. At the same time, the hit rate appeared sufficiently different from zero, suggesting our approach could discriminate hits while not being excessively restrictive. On balance, our primary repurposing screen confirms a reasonably predictable expectation: in aggregate, approved medications do not elicit anti-cancer effects, while a modest collection of drugs may exhibit useful pharmacology in combination with standard chemotherapy (Figure 1D).

**Table 1:** Primary Screen Hits.

Chemical Name	Brand Name	Target/Mechanism	Indication	Dose (mg/kg)	Route	Effect Size^a^	pValue^b^
Docetaxel	Taxotere	Microtubule anti-mitotic	Cancer	30	iv	0.08058	0.0006
Sumatriptan	Imitrex, Zecuity	5HT1-D,B agonist	Migraine	50	ip	0.1049	0.0014
Quinethazone	Hydromox	Na/Cl transporter, thiazide diuretic	Hypertension	50	po	0.1227	0.0025
Bumetanide	Bumex, Burinex	Na/K/Cl, loop diuretic	Hypertension	30	po	0.1227	0.0025
Allopurinol	Zyloprim	Xanthine oxidase inhibitor	Gout	300	po	0.1583	0.0072
Amlodipine	Norvasc	Calcium channel blocker	Hypertension	10	ip	0.1583	0.0072
Terbinafine	Lamisil	Fungal sqaulene epoxidase inhibitor	Antifungal	50	ip	0.1688	0.0111
Azathioprine	Azasan	GPAT, purine synthesis inhibitor	Immunosuppressant	10	ip	0.1782	0.0114
Tizanidine	Zanafex	Alpha2, adrenergic agonist	Muscle relaxant	12	po	0.2028	0.0178
Cefdinir	Omnicef	Cephalosporin	Antibiotic	10	po	0.2483	0.0346
Cyclosporin A	Neoral	Cyclophilin/Calcineurin Inhibitor	Immunosuppressant	40	ip	0.2451	0.0348
Paracetamol	Tylenol	NSAID COX enzyme inhibitor	Pain	300	ip	0.2506	0.0482
Cefepime	Maxipime	Cephalosporin	Antibiotic	100	ip	0.2722	0.0675
Candesartan	Atacand	Angiotensin receptor blocker	Hypertension	10	ip	0.1848	0.0727
Etoposide	Etopophos	Topoisomerase inhibitor	Cancer	10	ip	0.1848	0.0727
Dexlansoprazole	Kapidex	Proton pump inhibitor	Gastric reflux	150	ip	0.2941	0.0862

Hits significant in primary screen survival analysis are shown; near-neighbors in the tail of the screening hit distribution are shown, additionally.

a. Mantel-Hanzel hazard ratio, combo treatment compared to 5 mg/kg temozolomide alone.

b. Chi-square, survival analysis.

We performed confirmation studies for 33 drugs from the primary screen that were not known chemotherapeutics (Figure S1). Eight of eleven (73%) candidates that exhibited statistical significance in the primary screen were confirmed in a second test. We also queried 22 additional drugs that did not show significance initially. As anticipated, fewer (3 of 22; 14%) of these agents demonstrated efficacy in the secondary screen if they were negative in the primary screen, suggesting another degree of validation for our experimental approach. As an additional confirmatory step, we dosed eight of the primary hits in the absence of temozolomide (Figure S1). As may be expected, none of these safe drugs showed primary anti-cancer effects, again suggesting that our screen revealed novel drugs with potential anti-tumor effects only in the background of standard chemotherapy.

**Figure 2:**
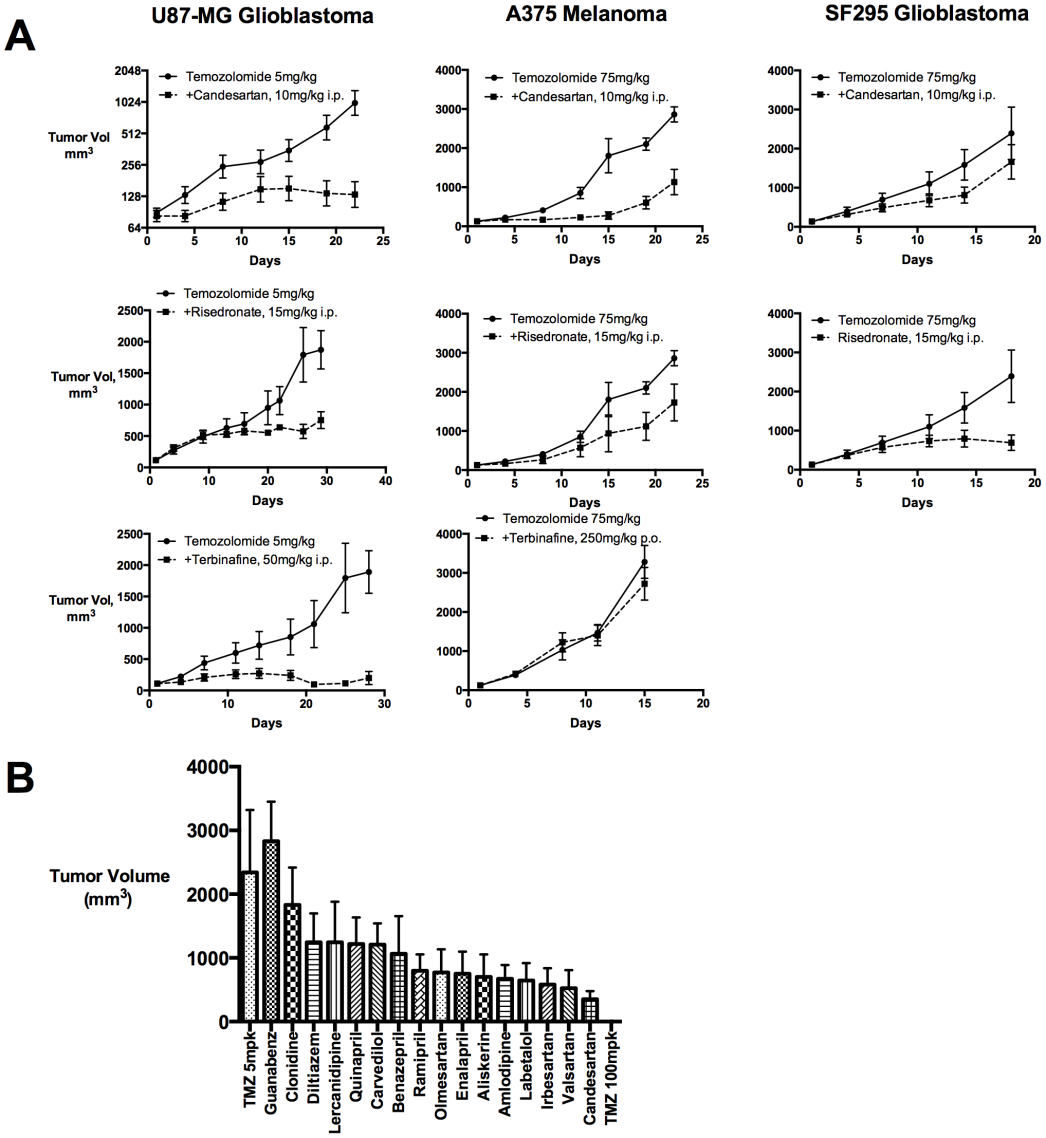
Screen validation and confirmation. A. Hit validation in additional models. Selected primary screen hits were tested in combination with sub-efficacious doses of temozolomide in a model of glioblastoma and melanoma xenograft models. To assess pharmacological effects terbinafine was dosed p.o in A375 model at a dose interval five times greater than its screening dose administered by i.p. route. In primary screen comparisons, risedronate was significant by survival analysis at a cut-off at 1000 mm^3^, but not 500 mm^3^. B. Hypertension drug combinations. Drugs were dosed in combination in the U87-MG model, at the dose in the primary screen, or as indicated in supplemental materials. Effects on tumor growth were assessed at Day 20 of the study. Means plus SEM shown (N = 10); candesartan and temozolomide 100 mg/kg significant by M-ANOVA at p<0.05.

We further validated 15 confirmed hits in two additional xenograft models: the A375 melanoma and SF295 glioblastoma human cancer lines (Supplemental Methods, Figure 2). Six drugs demonstrated activity in one of these orthogonal models when dosed in combination with temozolomide. In summary, our screening strategy appears to have discovered several drugs that exhibit *bona fide* activity with the alkylating agent temozolomide across multiple tumor types.

Provided with some degree of confidence, we sought to further characterize our resultant hits. Because of its unbiased, empirical nature, our screen should reveal anti-cancer effects caused by a diversity of factors. First, combination drugs could modulate the intrinsic activity of temozolomide, by affecting either its primary activity of DNA alkalation, or by modifying consequent DNA damage responses including cell cycle arrest and apoptosis. Likewise, temozolomide and induced DNA alkylation could, in a neomorphic fashion, convert signaling induced by a combination drug into novel cell growth or cell survival signals. Furthermore, any and all of these scenarios could be the result of either cell autonomous or non-cell autonomous processes: the two agents could jointly target an isolated cancer cell, or induce autocrine or paracrine signaling to ultimately cause reduced tumor growth and survival. We explored some possible mechanisms of efficacy for one candidate drug of interest, the angiotensin-II-receptor blocker candesartan.

First, we assessed whether concomitant candesartan dosing simply altered the primary pharmacokinetics of temozolomide [17], [18]. We found candesartan did not significantly alter the biodistribution of temozolomide across several tissues, suggesting this repurposing candidate does not mediate its effects merely through drug metabolism interactions (Table S2 in File S1). Additionally, we tested whether candesartan directly increased the cellular toxicity of temozolomide in cultured U87MG cells. A fixed-ratio (1-to-2.4) combination was employed, starting at suprapharmacologic concentrations of 40 uM temozolomide and 16.7 uM candesartan, respectively. Across these concentrations, we noted limited *in-vitro* potency of combination candesartan and temozolomide (Figure S2), and therefore we turned back to *in-vivo* models to explore the nature of this pharmacological interaction.

Next, we assessed how other drugs with similar pharmacologic mechanisms would perform in the primary U87MG screening model. Interestingly, we found that multiple angiotensin II receptor inhibitor drugs (“-artans”) were also effective in combination with temozolomide (Figure 2b). Furthermore, additional drugs such as the renin inhibitor aliskerin, as well as calcium channel blockers (amlodipine) and ACE inhibitors (enalapril) also appeared marginally effective. These findings suggest several interesting conclusions: 1.) our primary screen results can be confirmed with drugs acting via similar mechanisms of action 2.) an unknown pharmacophore targeted by several angiotensin-II-receptor inhibitor scaffolds may possess anti-cancer pharmacology 3.) *in-vivo* screens may reveal complex biological activity in pathways that can be inhibited by diverse drugs.

Lastly, we assessed whether the combination of candesartan/temozolomide caused toxicity specifically in cancer tissues, or more generally altered the pharmacologic margin of temozolomide. We observed that, at high doses, candesartan combination decreased lymphocyte and red blood cell counts; these are two dose-limiting adverse events that affect the approved use of temozolomide in humans (Table S3 in File S1). Additionally, we noted that high-dose candesartan aggravated weight loss and other impaired constitutional signs of health in mice when dosed in combination (data not shown). These finding suggest two conclusions. First, while the additional efficacy conferred by the addition of candesartan may be desirable, the combination could also increase adverse events, resulting in a risk-benefit profile largely unchanged from temozolomide dosing alone. Second, the mechanistic pharmacology underlying our results is likely complex: candesartan may alter both intrinsic and secreted factors that cause decreased viability in both a cell-autonomous, and non-cell autonomous, fashion—this may lead to suboptimal cancer targeting. While beyond the scope of the investigation we report here, it is likely that additional preclinical studies may prove that our screen revealed novel and desirable therapeutic opportunities.

**Figure 3:**
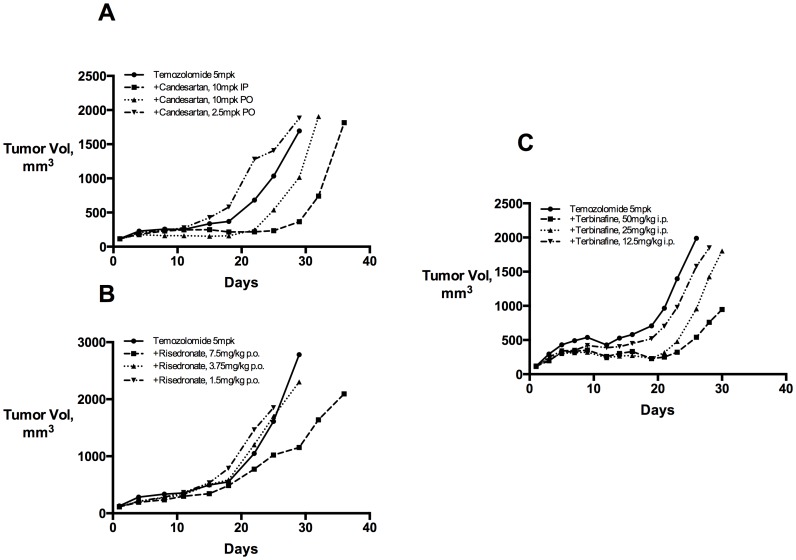
A. Candesartan dose range confirmation in the U87-MG model. Candesartan was confirmed at its screening dose and route; efficacy diminished following oral administration near and below the stated MTD dose equivalent of 14.3 mg/kg. B. Risedronate dose range confirmation. Risedronate exhibited modest combination effects when dosed orally at 7.5 mg/kg, which near MHRDD dose equivalent of 5 mg/kg (compare to screen result at 15 mg/kg, i.p.). The dose exposure trend was modest, and appeared equivalent at doses slightly below 5 mg/kg. C. Terbinafine dose range confirmation. Terbinafine was tested by i.p. administration. Efficacy diminished as drug was administered below the human dose equivalent of 4 mg/kg. Group means shown; cohort SEM not shown for clarity.

While mechanistic study of our screening hits may be useful, it could also be preferable to validate candidates directly through clinical testing. To rapidly translate our findings, it remained necessary to demonstrate efficacy at drug exposures nearer what is achieved in everyday human use. We used the prescribing labels for candesartan, the antifungal terbinafine, and the bisphosphonate risedrontate to select a range of oral doses for testing the U87MG model (methods). Compared to our screening efforts at maximum tolerated doses, the efficacy of each of these drugs decreased to insignificant levels as each was tested nearer to label-approved dose-equivalents (Figure 3). Broadly, these results imply that additional safety studies, both alone and possibly in combination with temozolomide, are required before these drug candidates could be tested in human cancer patients.

## Discussion

Several important conclusions are suggested by our efforts to discover drug repurposing candidates in cancer. First, despite their complexity, the *in-vivo* studies we conducted can identify numerous novel drug candidates by broad-based discovery screening across many tens, if not hundreds, of compounds. The hits exhibit pharmacology that appears consistent and reproducible across multiple model systems, and may suggest structure-activity and pathway relationships that were previously unknown and unexpected.

Our principle motivation in performing direct in-vivo, as opposed to primary cell combination screening, was to uncover drug activities in complex, multicellular contexts that are difficult to model using in-vitro systems. Encouragingly, we note that recently, additional groups have largely confirmed our findings regarding the interaction between angiotensin inhibition and chemotherapy. The authors propose a complex mode-of-action involving vasculogenesis in complex tumor stroma tissue [19]. An ongoing clinical trial (NCT01821729) may further confirm the therapeutic potential of the complex pharmacology discovered in our screens.

Despite these benefits of in-vivo screens, we also note several key limitations. Most importantly, it is assumed that repurposing screens identify drugs that can be rapidly tested for efficacy in clinical trials. To-date, our efforts have only yielded candidates that would require extensive preclinical and clinical safety studies before efficacy could be tested in patients. While this may seem to be the likely consequence from our “greedy” primary screens that tested near-maximum-tolerated doses, we highlight a converse risk: it seems likely our primary screen hit rate would have decreased to trivial or near-zero levels, had we assessed lower doses initially.

Taken in context, we suggest that in-vivo repurposing screens in the background of cancer therapy may be relatively resource-inefficient. As background, this study reflects the work of several team members who contributed significant efforts over approximately 18 months. Taken from a perspective of either committed funds or collective effort, the project consumed resources largely equivalent to those required to transition a single, novel drug from preclinical and clinical safety studies into efficacy studies in cancer patients. We have discovered novel pharmacology with potential use for cancer treatment, but also acknowledge that balancing the effort and value of repurposing screens versus other research priorities could prove difficult across the wider cancer research community.

## Supporting Information

Figure S1 **Monotherapy efficacy of candidate hits in U87-MG xenografts.** Compounds were administered at the same dose and route employed during the primary screen. Tumor measurements were assessed on Day 16. Group means are shown; error is SEM, N = 8–10.
(PPTX)

Figure S2 **Modest combination effects of temozolomide and candesartan in U87MG cells in-vitro.** U87MG cells were cultured in standard conditions (DMEM with 10% FBS), and exposed for 72 hours with compounds at various concentrations. The highest concentration of temozolomide employed was 300 uM, at least one-log above average therapeutic concentrations observed during clinical administration. Candesartan was tested at concentration ranges as high as 16.7 uM, again well beyond therapeutic drug exposures. Compounds were combined at a fixed dose ratio of 2.4 to 1 (e.g. 40 uM temozolomide to 16.7 uM candesartan). Cisplatin was employed as a cytotoxic control compound, at concentrations starting at 100 uM. Cell viability was assessed using colorimetric methods (MTS assays). Temozolomide showed minor viability effects in U87MG up to 300 uM in-vitro. The addition of candesartan up to 16.7 uM did not potently potentiate treatment with up to 40 uM temozolomide, compared to cisplatin, which induced prominent inhibition of cell viability.
(PPTX)

File S1 **Tables S1–S3.** Table S1. Phenix Cancer Library. Provided as Excel file in online supplemental materials. Table S2. Confirmation Studies in U87MG xenograft model. Mice bearing U87-MG were dosed with temozolomide in combination with the agents listed. Survival analysis was performed as described; metrics are provided for comparison. Table S3. Hematopoietic alterations by candesartan in combination with temozolomide. C57BLJ6 mice were dosed with temozolomide, with or without candesartan at the doses and routes indicated. Blood samples were collected for complete blood count hematology analysis. Unchanged parameters are not shown; cell populations showing significant changes (italicized) in multiple dose groups are shown below. * p <0.05 with ANOVA, Dunnett's post-hoc test. Values shown are group mean (SEM), N = 10.
(DOCX)
