# Supplementary figures and images for: Systematic Repurposing Screening in Xenograft Models Identifies Approved Drugs with Novel Anti-Cancer Activity
Source: PLoS One. 2014 Aug 5;9(8):e101708. doi: 10.1371/journal.pone.0101708 (PMC4122340; doi:10.1371/journal.pone.0101708)

## Slide 1
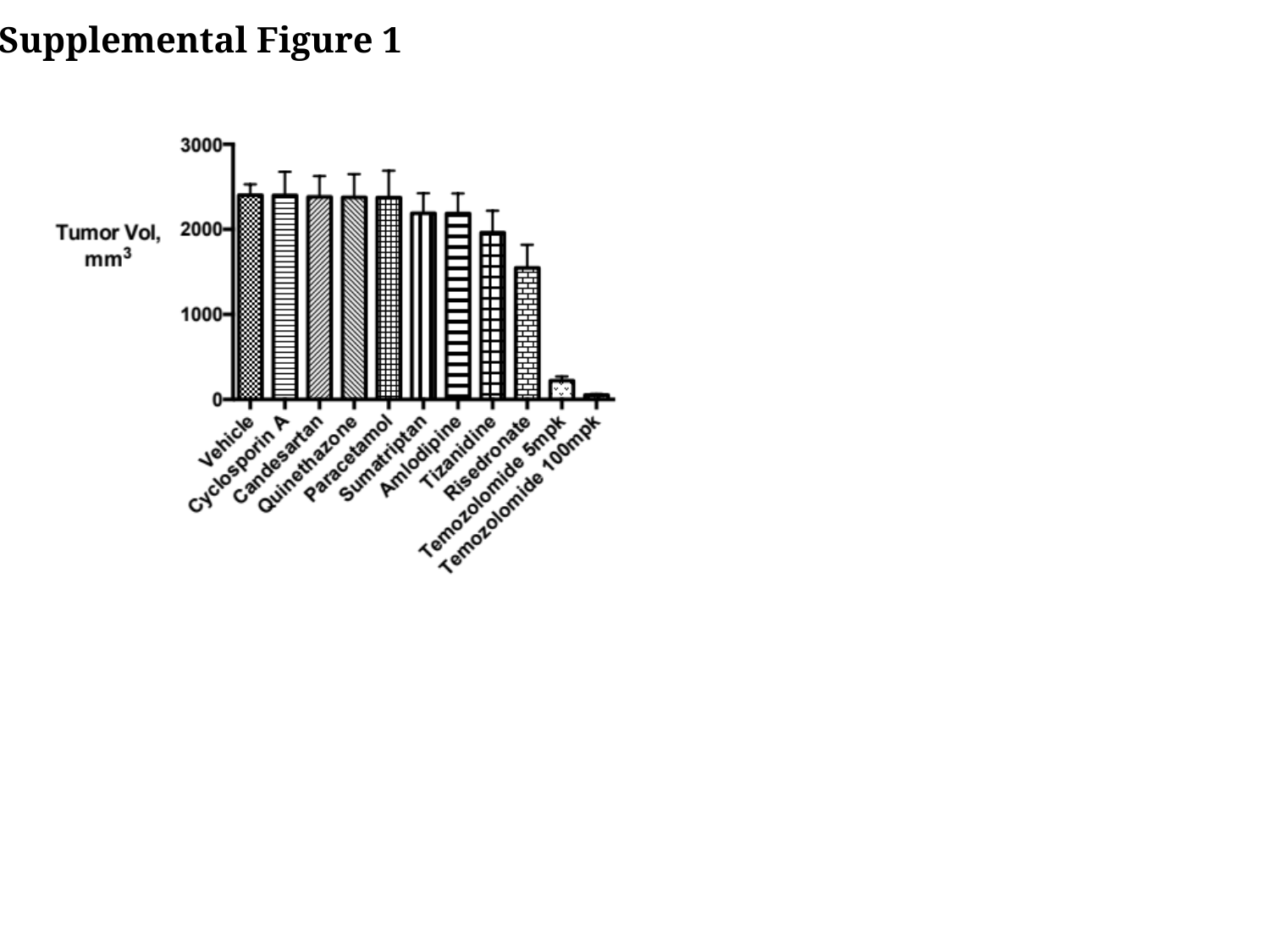

Supplemental Figure 1

Supplement: Figure S1 — Monotherapy efficacy of candidate hits in U87-MG xenografts. Compounds were administered at the same dose and route employed during the primary screen. Tumor measurements were assessed on Day 16. Group means are shown; error is SEM, N = 8–10. (PPTX) [file pone.0101708.s001.pptx]

## Slide 1
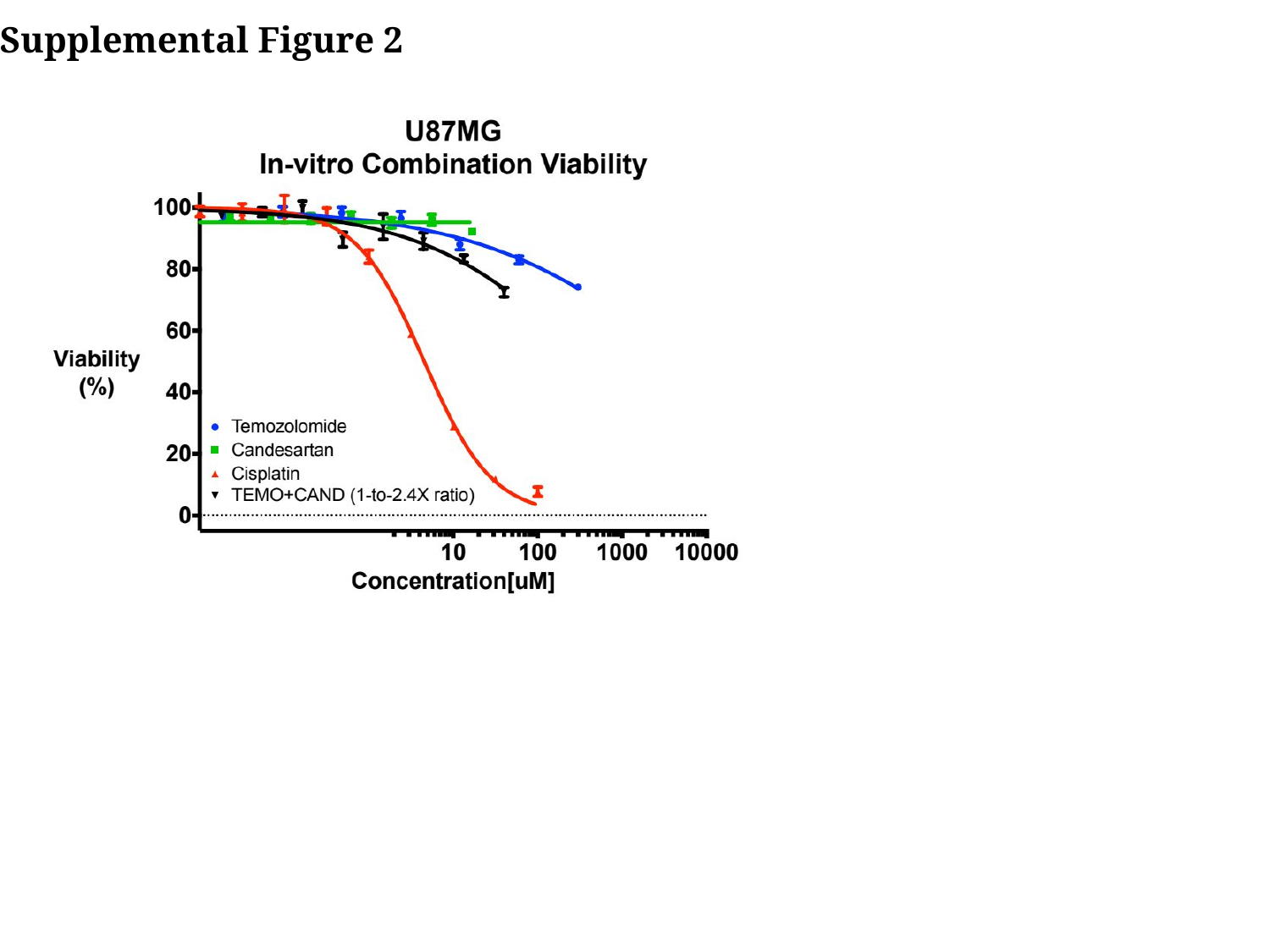

Supplemental Figure 2

Supplement: Figure S2 — Modest combination effects of temozolomide and candesartan in U87MG cells in-vitro. U87MG cells were cultured in standard conditions (DMEM with 10% FBS), and exposed for 72 hours with compounds at various concentrations. The highest concentration of temozolomide employed was 300 uM, at least one-log above average therapeutic concentrations observed during clinical administration. Candesartan was tested at concentration ranges as high as 16.7 uM, again well beyond therapeutic drug exposures. Compounds were combined at a fixed dose ratio of 2.4 to 1 (e.g. 40 uM temozolomide to 16.7 uM candesartan). Cisplatin was employed as a cytotoxic control compound, at concentrations starting at 100 uM. Cell viability was assessed using colorimetric methods (MTS assays). Temozolomide showed minor viability effects in U87MG up to 300 uM in-vitro. The addition of candesartan up to 16.7 uM did not potently potentiate treatment with up to 40 uM temozolomide, compared to cisplatin, which induced prominent inhibition of cell viability. (PPTX) [file pone.0101708.s002.pptx]
