# Supplementary material for: Systematic Repurposing Screening in Xenograft Models Identifies Approved Drugs with Novel Anti-Cancer Activity
Source: PLoS One. 2014 Aug 5;9(8):e101708. doi: 10.1371/journal.pone.0101708 (PMC4122340; doi:10.1371/journal.pone.0101708)
Supplement: File S1 — Tables S1–S3. Table S1. Phenix Cancer Library. Provided as Excel file in online supplemental materials. Table S2. Confirmation Studies in U87MG xenograft model. Mice bearing U87-MG were dosed with temozolomide in combination with the agents listed. Survival analysis was performed as described; metrics are provided for comparison. Table S3. Hematopoietic alterations by candesartan in combination with temozolomide. C57BLJ6 mice were dosed with temozolomide, with or without candesartan at the doses and routes indicated. Blood samples were collected for complete blood count hematology analysis. Unchanged parameters are not shown; cell populations showing significant changes (italicized) in multiple dose groups are shown below. * p <0.05 with ANOVA, Dunnett's post-hoc test. Values shown are group mean (SEM), N = 10. (DOCX) [file pone.0101708.s003.docx]

**Supplemental Online Information**

**Supplemental Table 1:** Phenix Cancer Library

Provided as Excel file in online supplemental materials

| **Chemical Name** | **Primary Screen, ChiSquare** | **Primary Screen, pValue** | **Primary Screen, Mantel HR** | **Replication Study, ChiSquare** | **Replication Study, pValue** | **Replication Study, Mantel HR** | **Significant in Both Screens?** |
| --- | --- | --- | --- | --- | --- | --- | --- |
| Sumatriptan | 10.22 | 0.0014 | 0.1049 | 5.308 | 0.0212 | 0.2439 | YES |
| Bumetanide | 9.117 | 0.0025 | 0.1227 | 5.767 | 0.0163 | 0.2279 | YES |
| Quinethazone | 9.117 | 0.0025 | 0.1227 | 6.007 | 0.0143 | 0.2426 | YES |
| Amlodipine besylate | 7.227 | 0.0072 | 0.1583 | 9.584 | 0.002 | 0.1484 | YES |
| Allopurinol | 7.227 | 0.0072 | 0.1583 | 1.476 | 0.2245 | 0.5243 | NO |
| Terbinafine | 6.442 | 0.0111 | 0.1688 | 7.691 | 0.0056 | 0.1767 | YES |
| Azathioprine | 6.397 | 0.0114 | 0.1782 | 0.7349 | 0.3913 | 0.6225 | NO |
| Tizanidine | 5.616 | 0.0178 | 0.2028 | 4.567 | 0.0326 | 0.2881 | YES |
| Cefdinir | 4.465 | 0.0346 | 0.2483 | 0.1143 | 0.7353 | 0.8289 | NO |
| Cyclosporin A | 4.453 | 0.0348 | 0.2451 | 6.864 | 0.0088 | 0.2131 | YES |
| Paracetamol | 3.902 | 0.0482 | 0.2506 | 10.58 | 0.0011 | 0.1259 | YES |
|  |  |  |  |  |  |  |  |
| Candesartan cilexetil | 3.222 | 0.0727 | 0.1848 | 12.48 | 0.0004 | 0.09904 | YES |
| Levetiracetam | 2.824 | 0.0929 | 3.647 | 2.937 | 0.0866 | 0.88 | NO |
| Pregabalin | 2.537 | 0.1112 | 0.3403 | 2.163 | 0.1414 | 0.4196 | NO |
| Clozapine | 2.326 | 0.1272 | 0.3403 | 0.02817 | 0.8667 | 0.9081 | NO |
| Leflunomide | 2.203 | 0.1378 | 0.3263 | 6.427 | 0.0112 | 0.233 | YES |
| Rasagiline | 1.741 | 0.1871 | 0.3753 | 0.7631 | 0.3824 | 0.5638 | NO |
| Glycyrrhizic acid | 1.337 | 0.2476 | 2.986 | 0.001215 | 0.9722 | 1.02 | NO |
| Loxapine | 1.2 | 0.2733 | 9.025 | 1.523 | 0.2171 | 0.4798 | NO |
| Atosiban | 0.6489 | 0.4205 | 1.863 | 3.391 | 0.0655 | 0.3514 | NO |
| Atovaquone | 0.5904 | 0.4423 | 1.787 | 0.2784 | 0.5977 | 1.32 | NO |
| Sapropterin | 0.3177 | 0.573 | 0.6591 | 1.453 | 0.228 | 0.4808 | NO |
| Scopolamine | 0.2009 | 0.654 | 1.442 | 1.032 | 0.3097 | 1.741 | NO |
| Montelukast | 0.185 | 0.6671 | 1.406 | 0.03485 | 0.8519 | 0.9043 | NO |
| Khellin | 0.1045 | 0.7464 | 1.285 | 0.3401 | 0.5598 | 1.391 | NO |
| Risedronate | 0.0737 | 0.786 | 0.8157 | 6.56 | 0.0104 | 0.225 | YES |
| Carvedilol | 0.0414 | 0.8388 | 0.8422 | 1.471 | 0.2252 | 0.5042 | NO |
| Eszopiclone | 0.02062 | 0.8858 | 1.119 | 1.597 | 0.2064 | 2.065 | NO |
| Mitoxantrone HCl | 0.009 | 0.9244 | 1.054 | 0.009 | 0.9244 | 1.054 | NO |
| Aprepitant | 0.007337 | 0.9317 | 1.076 | 0.7007 | 0.4025 | 1.53 | NO |
| Linezolid | 0.006971 | 0.9335 | 0.9327 | 0.03738 | 0.8467 | 1.113 | NO |
| Imiquimod | 0.003431 | 0.9533 | 1.053 | 0.00002446 | 0.9961 | 0.9973 | NO |
| Flutrimazole | 0.0009969 | 0.9748 | 1.026 | 0.2538 | 0.6144 | 1.332 | NO |

**Supplemental Table 2:** Confirmation Studies in U87MG xenograft model

Mice bearing U87-MG were dosed with temozolomide in combination with the agents listed. Survival analysis was performed as described; metrics are provided for comparison.

**Supplemental Table 2:** Temozolomide biodistribution in the presence of candesartan

Mice bearing U87-MG were dosed with C14-labeled temozolomide alone, or together with candesaratan for two days. Compound concentrations in blood and tumor samples, and the ratio of compound accumulation, are shown following repeated dosing. Statistical comparisons were made on an AUC basis for compound levels, and by t-test analysis for accumulation ratios at each time point. Values shown are means with SEM in parentheses (N=4).

| **Time, Post-Dose (min)** | **Blood (ug/mL)** | | **Tumor (ug/g)** | | **Tumor: Blood Ratio** | |
| --- | --- | --- | --- | --- | --- | --- |
|  | Temozolomide5mg/kg | +Candesartan  10mg/kg i.p. | Temozolomide5mg/kg | +Candesartan 10mg/kg i.p. | Temozolomide 5mg/kg | +Candesartan 10mg/kg i.p. |
| 30 | 1.06 (0.30) | 1.40 (0.45) | 1.25 (0.17) | 0.56 (0.04) | 140.1% (29.5) | 67.9% (24.3) |
| 60 | 1.07 (0.35) | 2.23 (0.23) | 1.10 (0.14) | 1.71 (0.51) | 135.6% (37.7) | 84.0% (29.7) |
| 90 | 0.76 (0.02) | 1.56 (0.34) | 0.97 (0.25) | 1.15 (0.34) | 128.1% (32.0) | 86.5% (35.5) |

**Supplemental Table 3:** Hematopoietic alterations by candesartan in combination with temozolomide.

C57BLJ6 mice were dosed with temozolomide, with or without candesartan at the doses and routes indicated. Blood samples were collected for complete blood count hematology analysis. Unchanged parameters are not shown; cell populations showing significant changes (italicized) in multiple dose groups are shown below. * p <0.05 with ANOVA, Dunnett’s post-hoc test. Values shown are group mean (SEM), N=10.

| **Day 6 Post Dosing** | | | |
| --- | --- | --- | --- |
|  |  |  |  |
| **Dose Group** | **RBC (1x10^6^/mL)** | **WBC (1x10^3^/mL)** | **Lymphocytes (1x10^3^/mL)** |
|  |  |  |  |
| **Temozolomide, 25mg/kg** | 10.3 (0.15) | 7.52 (0.56) | 5.71 (0.42) |
| **+Candesartan, 10mg/kg, i.p.** | *8.34 (0.16)** | *5.23 (0.49)** | *3.77 (0.36)** |
| **+Candesartan, 10mg/kg, p.o.** | *9.50 (0.14)** | *5.80 (0.54)** | *4.35 (0.43)** |
| **+Candesartan, 5mg/kg, p.o.** | *9.51 (0.09)** | 6.48 (0.43) | 5.03 (0.35) |
| **+Candesartan, 2.5mg/kg,p.o.** | 9.69 (0.12) | 6.02 (0.29) | 4.71 (0.25) |
| **+Candesartan, 1.25mg/kg, p.o.** | 9.76 (0.10) | 7.00 (0.45) | 5.43 (0.32) |
| **Day 10 Post Dosing** | | | |
|  |  |  |  |
| **Dose Group** | **RBC (1x10^6^/mL)** | **WBC (1x10^3^/mL)** | **Lymphocytes (1x10^3^/mL)** |
|  |  |  |  |
| **Temozolomide, 25mg/kg** | 8.3 (0.17) | 8.19 (0.34) | 5.23 (0.30) |
| **+Candesartan, 10mg/kg, i.p.** | *7.10 (0.30)** | 7.44 (1.0) | 4.25 (0.48) |
| **+Candesartan, 10mg/kg, p.o.** | 7.92 (0.10) | 8.05 (0.40) | 5.06 (0.31) |
| **+Candesartan, 5mg/kg, p.o.** | 8.37 (0.08) | 7.68 (0.80) | 5.00 (0.42) |
| **+Candesartan, 2.5mg/kg,p.o.** | 7.90 (0.16) | 6.77 (0.65) | 4.44 (0.34) |
| **+Candesartan, 1.25mg/kg, p.o.** | 7.95 (0.17) | 8.45 (0.62) | 5.67 (0.45) |
